# Combined influence of quantum iterative reconstruction level and kernel sharpness on image quality in photon counting CT angiography of the upper leg

**DOI:** 10.1038/s41598-024-79188-3

**Published:** 2024-11-13

**Authors:** Kristina Krompaß, Florian Andreas Goldbrunner, Viktor Hartung, Süleyman Ergün, Dominik Peter, Robin Hendel, Henner Huflage, Theresa Sophie Patzer, Jan-Lucca Hennes, Thorsten Alexander Bley, Jan-Peter Grunz, Philipp Gruschwitz

**Affiliations:** 1 Department of Diagnostic and Interventional Radiology, University Hospital of Würzburg, Oberdürrbacher Str. 6, 97080 Würzburg, Germany; 2 Institute of Anatomy and Cell Biology, University of Würzburg, Würzburg, Germany; 3 Department of General, Visceral, Transplant, Vascular, and Pediatric Surgery, University Hospital of Würzburg, Würzburg, Germany; 4 Department of Radiology, University of Wisconsin – Madison, Madison, WI 53792 USA

**Keywords:** Preclinical research, Computed tomography

## Abstract

Aim was to evaluate the influence of different quantum iterative reconstruction (QIR) levels on the image quality of femoral photon-counting CT angiographies (PCD-CTA).

Ultra-high resolution PCD-CTA were obtained from both extremities of five extracorporeally-perfused cadavers using constant tube voltage and maximum radiation dose (71.2 ± 11.0 mGy). Images were reconstructed with three kernels (Bv48, Bv60, Bv76) and the four available levels of QIR. Signal attenuation in the arterial lumen, muscle, and fat were measured. Contrast-to-noise ratios (CNR) and blurring scores were calculated for objective assessment. Six radiologists evaluated the subjective image quality using a pairwise comparison tool.

Higher QIR level resulted in a decisive image noise reduction, especially with sharper convolution kernels (Bv60: Q1 11.5 ± 6.3 HU vs. Q4 8.4 ± 2.6 HU; *p* < 0.001). Largest improvement of CNR was recorded with ultra-sharp reconstructions (Bv76: Q1 20.2 ± 4.4 vs. Q4 28.0 ± 3.5; *p* < 0.001). Blurring decreased with higher QIR levels for soft Bv48, remained constant for medium Bv60, and increased for sharp Bv76 reconstructions. Subjective QIR level preference varied kernel depending, preferred combinations were: Bv48/Q4, Bv60/Q2, Bv76/Q3. Interrater agreement was excellent.

Sharp kernels benefited most from noise reduction of higher QIR levels in lower extremity PCD-CTA. In sum, QIR level 3 provided the best objective and subjective image quality results.

**Supplementary Information:**

The online version contains supplementary material available at 10.1038/s41598-024-79188-3.

## Introduction

Computed tomography angiography (CTA) constitutes the primary diagnostic method for the peripheral arterial runoff, particularly in the acute setting, due to its widespread availability and short examination time^1,2^. The exclusion of an acute vascular occlusion and the graduation of peripheral arterial disease are decisive for evaluating further treatment options^3^. CTA reaches its technical limits with conventional energy-integrating detectors, particularly in the narrow-caliber vessels of the lower leg and in cases of high-grade calcification^4–7^. Photon-counting detector CT (PCD-CT) has the potential to overcome these problems at least in part through intrinsic noise reduction and higher spatial resolution^8,9^. However, in addition to the detector-based improvements and the scanning protocol used, the reconstruction of raw data also has a key influence on the resulting image quality^10,11^. Besides selecting a reconstruction kernel optimized for the desired application, image-based noise reduction measures are of primary interest. Filtered back projection has been replaced by iterative image reconstruction approaches for these purposes in the last decade^12^. A special quantum iterative reconstruction algorithm was developed for the novel detector type (QIR, Siemens Healthineers, Forchheim, Germany) with the aim to overcome the preexisting limitations regarding data complexity and noise structure^13^. The vendor-specific fourth-generation algorithm provides four available levels referred as QIR 1–4 with increasing strength due to an increasing number of iterative reconstruction loops^13^. While the effect of different QIR choices has been previously studied for bone and abdominal imaging, their impact in lower extremity CTA has not been thoroughly investigated thus far^13,14^.

Therefore, the aim of this study was to evaluate the influence of different QIR levels in combination with vascular convolution kernels on the objective and subjective image quality in femoral PCD-CTA using an extracorporeally perfused human in-vitro model.

## Materials and methods

### Body donors and human perfusion model

Five fresh-frozen body donors were provided by the local anatomical institute for this study. After surgical preparation, conventional angiographic introducer sheaths were inserted in inguinal and infragenicular positions into the common femoral artery and popliteal artery. The arterial lower extremity runoff (of the upper thigh including the femoral popliteal artery) was perfused with an iodine-saline mixture using a peristaltic pump. Subsequently, CT angiographies were performed under continuous extracorporeal perfusion. Detailed information regarding the establishment of the model can be found elsewhere^15^.

### Ethical approval

Written informed consent from all body donors was obtained during their lifetime to use their bodies for research and science. The ethics committee of the University of Würzburg approved this study and waived the need for further consent (protocol number: 20220413 01). Used methods were carried out in accordance with relevant guidelines, regulations and in compliance with the requirements of the Declaration of Helsinki.

### CT scan protocol

CTA examinations were conducted with a first-generation PCD-CT (Naeotom Alpha, Siemens Healthineers). All scans were performed with a fixed tube voltage of 120 kV and the maximum tube current of 750–1100 mAs resulting in volume CT dose index of 59.9–88.2 mGy using the ultra-high resolution scan mode without pixel binning and a collimation of 120 × 0.2 mm. Of note, the large variation in the tube current and the radiation dose are based on deviations in the scan length and limitations in the total tube current due to scanner-side dose modulation (CareDose, Siemens). Both the helical pitch factor (0.2) and the gantry rotation time (1.0 s) were kept constant.

### Reconstruction and image parameters

Images were reconstructed for each leg with a separate field of view of 150 × 150 mm using three different vascular convolution kernels (Bv; Body-vascular) with different spatial frequencies (ρ_50_ equals the spatial frequency at 50% of the modulation transfer function): Bv48 (ρ_50_ = 5.40 lp/cm), Bv60 (ρ_50_ = 8.79 lp/cm), and Bv76 (ρ_50_ = 16.47 lp/cm). Slice thickness and increment were preset as 1.0 mm, respectively. The pixel matrices were selected by the scanner depending on the combination of spatial resolution and kernel: Bv40 to Bv60 with 512 × 512 pixels and Bv76 with 756 × 756 pixels. Only polychromatic images, which provide an intrinsic reduction of electronic noise in the energy spectrum below 20 keV, were analyzed. All image stacks were reformatted using the four applicable QIR levels (Q1 to Q4).

### Objective image quality

To determine the objective image quality, manual measurements of signal attenuation in Hounsfield units (HU) were performed with equal-sized regions of interest (30 mm^2^ or as large as possible) in the arterial lumen (HU_artery_) and surrounding muscle tissue (HU_muscle_) in each of the reconstructed image stacks on the same axial slice. Image noise was defined as the standard deviation (SD) of attenuation in subcutaneous fat tissue (SD_fat_). Measurements on four consistent planes of the thigh (proximal, middle, and distal superficial femoral artery as well as popliteal artery) were performed by a radiologist with 1 year of experience in CTA imaging using a clinical image archiving and communication system (Merlin, Phönix-PACS, Freiburg, Germany). Contrast-to-noise-ratios (CNR) were calculated with the formula $\:CNR=\:\frac{({HU}_{artery}-\:{HU}_{muscle)}}{{SD}_{fat}}$.

The image blur was objectified by calculating a reference value-free image blur matrix according to Crete et al.^16^ including all pixels of each analyzed image with the limit values “0” for no blur and “1” maximum blurring.

### Subjective image quality

Subjective image quality was evaluated using an in-house programmed, forced-choice pairwise comparison software. For this purpose, a representative image slice of each thigh was defined at the level of the mid superficial femoral artery and images of each kernel and QIR level pairing were exported (three kernels × four QIR levels) resulting in twelve combinations. Subsequently, images were presented in pairs within the browser-based comparison software to the six raters with 1 to 8 years of experience (12 × (12 − 1) / 2 = 66 pairs × 10 extremities = 660 choices). For each choice, raters were forced to make a decision for the subjectively higher quality image focusing primarily on vessel assessment. The decision-making process using the forced-choice pairwise comparison software is schematically depicted in the Supplementary Fig. S1.

### Statistics and data analysis

Dedicated software (DATAtab e.U., Graz, Austria) was utilized for statistical analysis. Metric-scaled data are given as mean value and standard deviation, while ordinal-scaled items are presented with median value and interquartile range. Significance was assumed for any p-level ≤ 0.05. Parameters of objective and subjective image quality were compared using Friedman’s rank test. Interrater-agreement was determined using Kendall’s concordance coefficient (W). Boxplots and bump charts were generated for visualization purposes. Data of the subjective image quality assessment were computed in a Jupyter Notebook environment using Python (V 3.8.15) and additional freely available libraries. In terms of subjective image quality, a Bradley-Terry model was used in reversed direction. Preference ranks from 1 (best) to 12 (worst) were determined for the individual rater and the whole group.

## Results

### Objective image quality

Slight variations in attenuation, as presented in Table 1, were observed within the arterial lumen and muscle tissue among the three kernels used. In addition, the QIR level also had a minimal effect on the absolute attenuation values (e.g., Bv76: Q1 398.0 ± 28.9 HU vs. Q4 400.7 ± 28.8 HU; *p* < 0.001; corresponds to 0.7%). Regardless of kernel selection, a higher QIR level resulted in a reduction of image noise (e.g., Bv60: Q1 11.5 ± 6.3 HU vs. Q4 9.5 ± 2.3 HU; *p* < 0.001). Both the absolute reduction in noise and the noise difference between the individual QIR levels were larger for sharper convolution kernels (Bv76: Q1 19.3 ± 3.2 HU vs. Q4 13.9 ± 1.1 HU; *p* < 0.001) than for softer ones (Bv48: Q1 7.1 ± 2.4 HU vs. Q4 6.6 ± 3.2 HU; *p* = 0.001). Of note, the noise reduction realized by selecting the next higher QIR level was only significant for reconstructions using Bv60 and Bv76.

**Table 1:** Overview of the attenuation values, image noise and contrast-to-noise-ratio values for all QIR level (Q1 to 4) depending on the convolution kernel used (Bv48, Bv60 and Bv76). Bv = body vascular; CNR = contrast-to-noise-ratio; HU = hounsfield units; QIR = quantum iterative reconstruction; SD = standard deviation.

Kernel	QIR level	Luminal HU (mean ± SD)	Muscle HU (mean ± SD)	SD (noise) fat (mean ± SD)	CNR
Bv48	Q 1	397.7 ± 29.7	50.0 ± 3.7	7.1 ± 2.4	59.7 ± 20.7
	Q 2	398.6 ± 29.8	50.2 ± 3.6	6.9 ± 2.8	66.0 ± 26.7
	Q 3	399.1 ± 29.9	50.3 ± 3.7	6.8 ± 3.0	70.6 ± 32.7
	Q 4	399.5 ± 30.0	50.4 ± 3.7	6.6 ± 3.2	75.0 ± 39.2
Bv60	Q 1	397.7 ± 29.0	49.5 ± 7.6	11.5 ± 6.3	38.2 ± 10.1
	Q 2	398.6 ± 29.0	50.9 ± 3.6	9.5 ± 2.3	43.8 ± 11.1
	Q 3	399.1 ± 28.9	51.1 ± 3.6	8.9 ± 2.4	47.4 ± 13.1
	Q 4	399.6 ± 29.0	51.4 ± 3.7	8.4 ± 2.6	51.6 ± 17.1
Bv76	Q 1	398.0 ± 28.9	50.0 ± 2.9	19.3 ± 3.2	20.2 ± 4.4
	Q 2	399,2 ± 28.8	50.4 ± 2.2	17.1 ± 2.2	22.8 ± 4.1
	Q 3	400.0 ± 28.8	50.6 ± 1.8	15.0 ± 1.4	25.9 ± 3.8
	Q 4	400.7 ± 28.8	50.8 ± 1.7	13.9 ± 1.1	28.0 ± 3.5

**Fig. 1:**
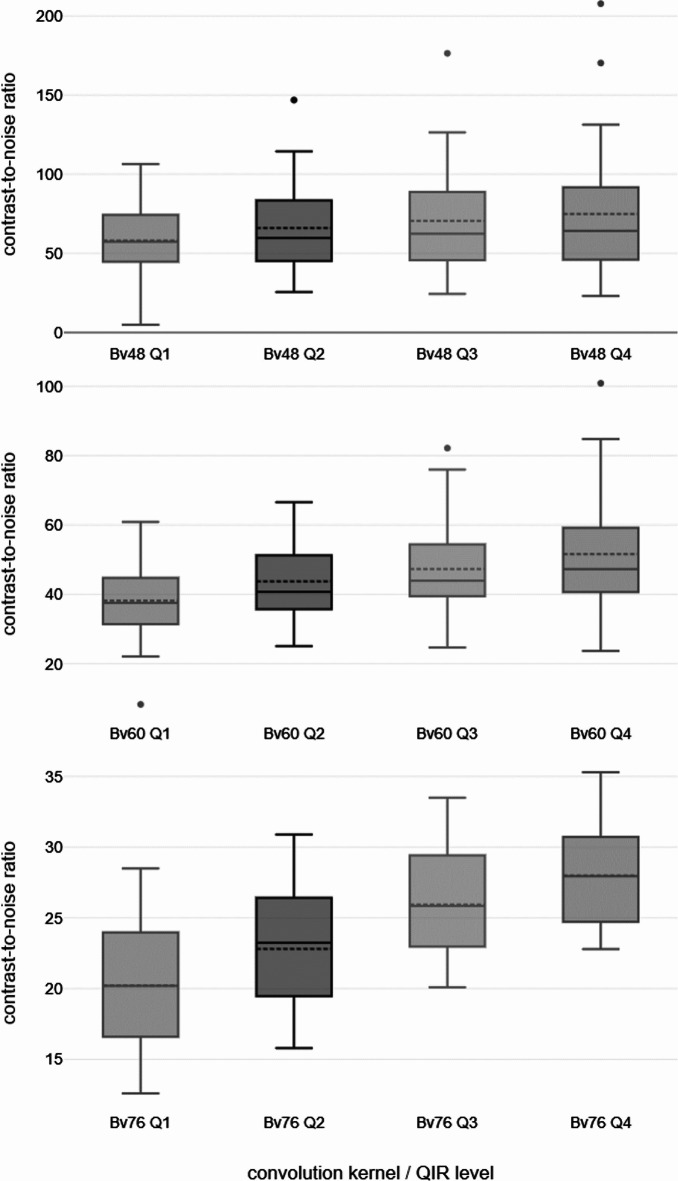
Contrast-to-noise ratios of each convolution kernel–QIR level combination. Top row: Bv48, middle row: Bv60, bottom row: Bv76; QIR levels 1 (left) to 4 (right). Bv = body vascular; QIR = quantum iterative reconstruction. The box shows the interquartile range (1st quartile 25% and 3rd quartile 75%). The 1.5-fold interquartile range defines the whiskers and the dots show the outliers between the 1.5-fold and the 3-fold interquartile range.

Since the mean HU values of the arterial lumen and muscle tissue are only marginally influenced by the QIR level, the CNR depends largely on image noise (SD_fat_). CNR therefore increased with higher QIR levels. The increase of CNR between the individual QIR levels was significant except for Q1 vs. Q2 and Q3 vs. Q4 at Bv48. Figure 1 shows the CNR values depending on the kernel and the QIR level used. Corresponding p-values can be found in Table 2. Images processed with Q1 provided between 73% and 79.6% of the CNR that Q4 images reached, so overall CNR rises with the QIR level.

**Table 2:** Pairwise comparison of objective image quality criteria (p-values). This table shows the p-values for the differences in the CNR for all QIR level (Q1 to 4) depending on the convolution kernel used (Bv48, Bv60 and Bv76). Significance level was indicated as: ^n.s^. = non-significant; ^#^*p* ≤ 0.050; ^##^*p* ≤ 0.010 and ^###^*p* ≤ 0.001. Bv = body vascular; CNR = contrast-to-noise-ratio; HU = hounsfield units; QIR = quantum iterative reconstruction; SD = standard deviation.

Kernel	QIR level	Luminal HU	Muscle HU	SD (noise) fat	CNR
Bv48	Q 1 vs. 2	0.090^n.s^.	0.401^n.s^.	0.999^n.s^.	0.999^n.s^.
	Q 2 vs. 3	0.028^#^	0.178^n.s^.	0.070^n.s^.	0.032^#^
	Q 3 vs. 4	0.014^#^	0.805^n.s^.	0.945^n.s^.	0.945^n.s^.
	Q 1 vs. 4	< 0.001^###^	< 0.001^###^	0.002^###^	0.001^###^
Bv60	Q 1 vs. 2	0.037^#^	0.037^#^	0.010^##^	0.010^##^
	Q 2 vs. 3	0.016^#^	0.003^##^	0.025^#^	0.028^#^
	Q 3 vs. 4	0.028^#^	0.080^n.s^.	0.019^#^	0.014^#^
	Q 1 vs. 4	< 0.001^###^	< 0.001^###^	< 0.001^###^	< 0.001^###^
Bv76	Q 1 vs. 2	0.032^#^	0.004^##^	0.004^##^	0.004^##^
	Q 2 vs. 3	0.143^n.s^.	0.003^##^	0.003^##^	0.004^##^
	Q 3 vs. 4	0.090^n.s^.	0.002^##^	0.008^##^	0.004^##^
	Q 1 vs. 4	< 0.001^###^	< 0.001^###^	< 0.001^###^	< 0.001^###^

**Fig. 2:**
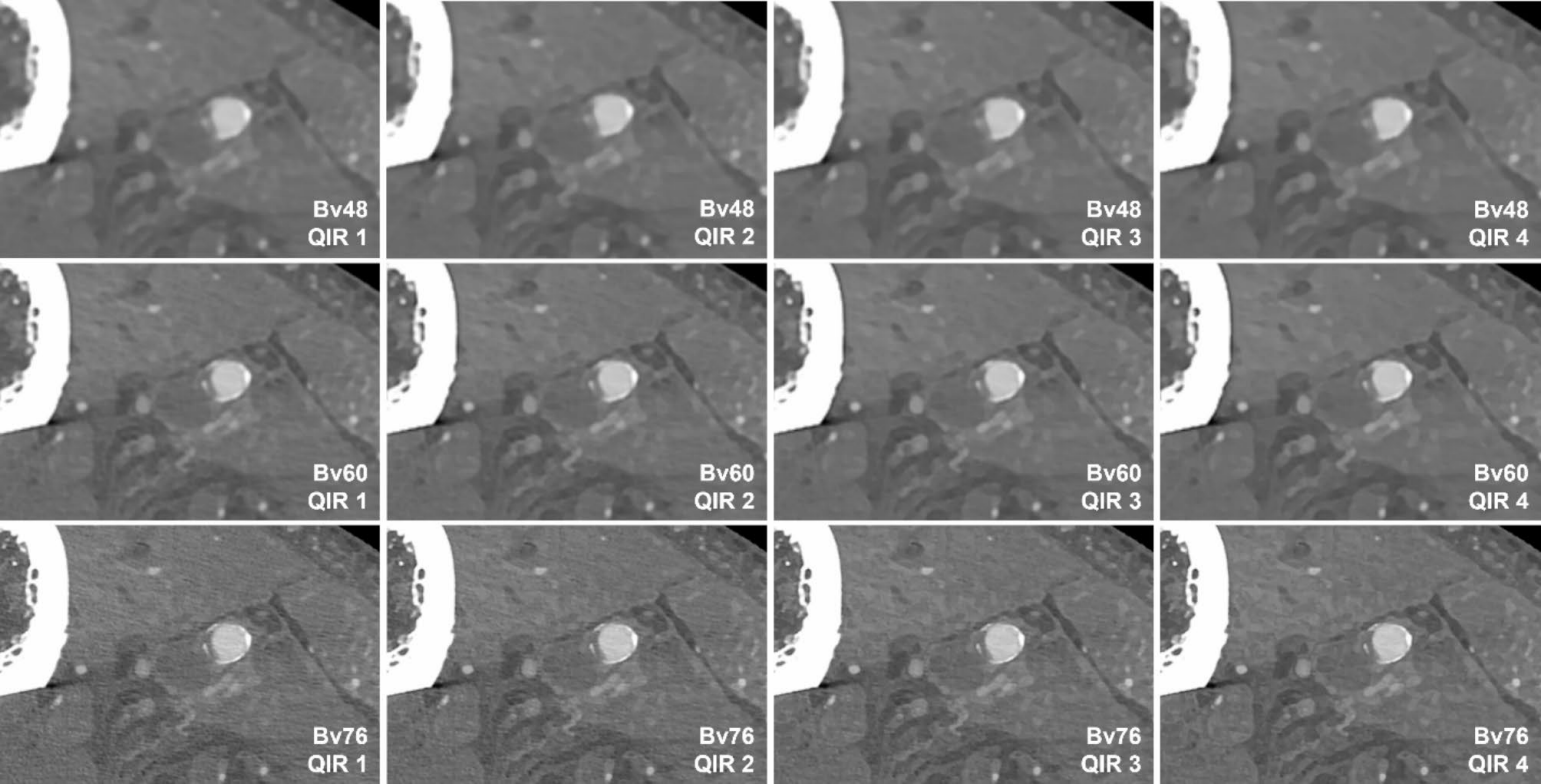
Image quality with each convolution kernel–QIR level combination. Images of the right leg of one body donor (middle superficial femoral artery). Top row: Bv48, middle row: Bv60, bottom row: Bv76. QIR level 1 to 4 from left to the right. Bv = body vascular; QIR = quantum iterative reconstruction.

The calculation of the blur metrics as another objective image quality marker showed divergent results depending on the used convolution kernel. While blurring decreased with higher QIR level for the soft Bv48, the calculated blur remained constant for images reconstructed with medium Bv60 and even increased in reconstructions reformatted with the sharp Bv76. Blur metrics are visualized in Table 3. The corresponding image impression is shown in Fig. 2 to facilitate direct comparison of the different reconstructions (Q1 – Q4, Bv48, Bv 60 and Bv78 for each QIR level).

**Table 3:** Image blurring metrics. Summary of the image blurring level depending on level of used quantum iterative reconstruction level and convolution kernel. 0 = no blur, 1 = maximum blur. Bv = body vascular; QIR = quantum iterative reconstruction.

	Bv48	Bv60	Bv76
**Q 1**	0.649	0.538	0.366
**Q 2**	0.636	0.535	0.380
**Q 3**	0.632	0.538	0.396
**Q 4**	0.627	0.539	0.405

### Subjective image quality

**Fig. 3:**
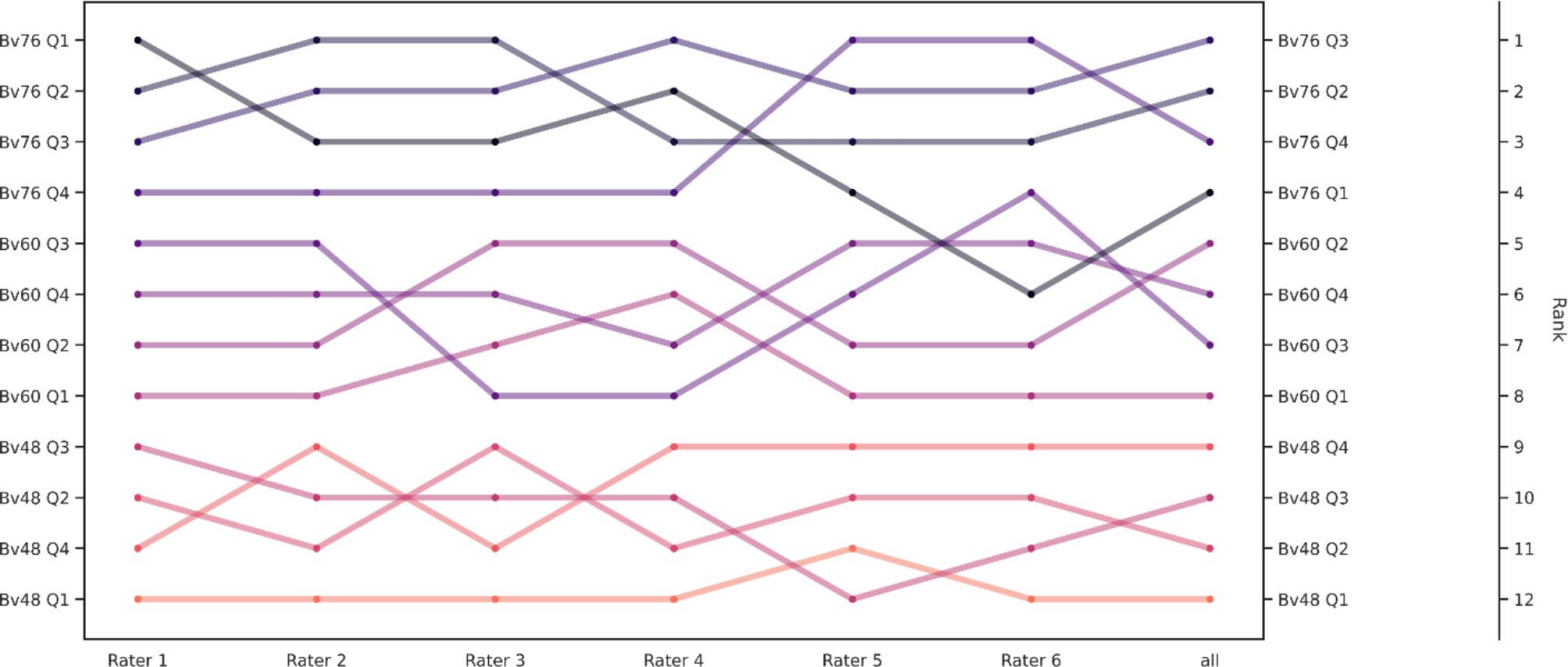
Bump Chart of subjective image quality assessment. The bump chart visualizes the individual ratings of each radiologist (raters 1 to 6) and the column on the left combines the results of all raters to an overall pooled subjective rating (marked with “all**”** in the bottom line) with ranks 1 and 12 indicating the best and worst image quality, respectively.

**Fig. 4:**
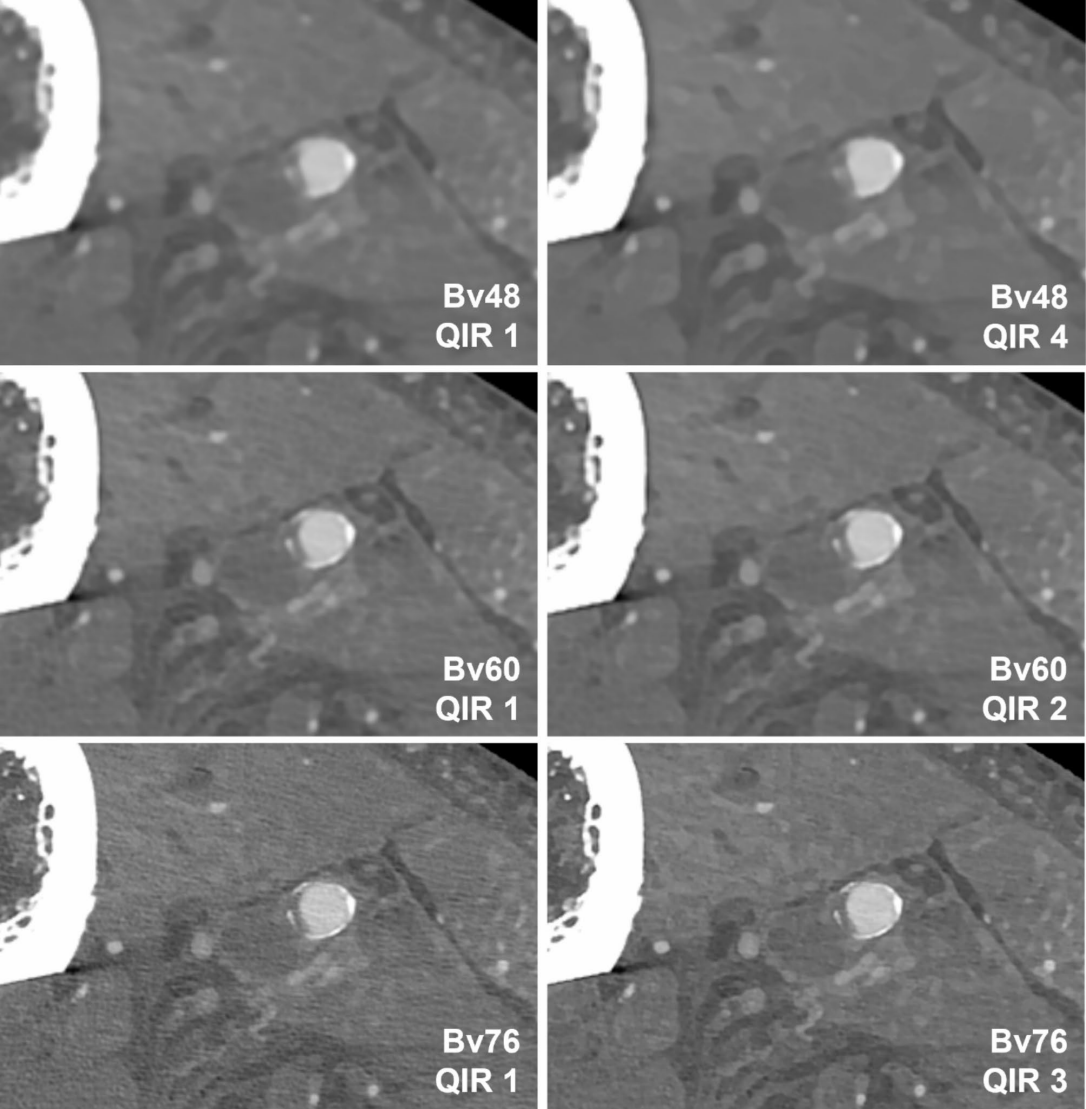
Direct comparison of the best and worst rated reconstructions. Representative images of the right leg of one body donor (middle superficial femoral artery). Top row: Bv48, middle row: Bv60, bottom row: Bv76. QIR level 1 to 4 from left to the right.

Sharper kernels were preferred over softer ones by all raters. While there was no unanimous consensus for the subjectively preferred QIR level, interrater agreement was very high with a Kendall W of 0.953 (*p* < 0.001). Bv48 Q1 was rated the worst and Bv76 Q3 the best combination overall. Looking at the kernels individually, Bv48 Q4, Bv60 Q2, and Bv76 Q3 achieved the best subjective ratings. An overview of the individual ratings and the pooled results of all raters are shown in Fig. 3, whereas Fig. 4 provides a side-by-side comparison of the images that achieved either the highest or lowest rating score.

## Discussion

This study investigated the influence of a dedicated quantum iterative reconstruction (QIR) algorithm on objective and subjective image quality in photon-counting detector CT angiographies of the upper leg arteries. Using an established in-vitro human perfusion model, we were able to show that a higher QIR level leads to an improvement of image quality in terms of image noise and contrast-to-noise-ratio. This influence is kernel dependent and stronger for sharper kernels. While subjective image quality can also be improved by selecting higher QIR levels, intermediate iterative reconstruction level were preferred for sharper kernels (Bv60: QIR 2, Bv76: QIR 3).

However, it is challenging to transfer iterative reconstruction algorithms originally designed for conventional energy-integrating detector CT to PCD-CT due to a more complex data structure. Variations in image acquisition, such as multi-energetic information, additional detectors as well as differences in the noise model need to be addressed for the optimal use of iterative reconstruction in PCD-CT. Furthermore, data reconstruction and material decompensation are usually performed in separate steps, which leads to a loss of image information. To offset this limitation, it is possible to perform image reconstruction and material decompensation in combined fashion^17^, however, this approach extends the average clinical reconstruction time by far. All in all, there is a high demand for specific algorithms suitable for PCD-CT in clinical routine^14^. While the influence of QIR on image quality has only been investigated in few studies and not for lower extremity CTA in particular, our results are generally consistent with those of other authors. Sartoretti et al. examined ultra-high resolution images in low-dose lung imaging and concluded that QIR level 3 provides the best results^18^. Another study from the same group could show that QIR level 4 is superior for assessing hypodense liver areas^14^. A recent study by Graafen et al. compared different QIR levels in pathologically transformed liver parenchyma, stating that higher QIR levels benefit the imaging of hepatocellular carcinoma. In this investigation, the highest CNR and a 50% reduction of the median noise level could be achieved with QIR level 4 without compromising image sharpness substantially^19^. Nonetheless, it is important to emphasize that the optimal QIR level strongly depends on the examined body region. In contrast to the aforementioned studies, which all focused on different types of soft tissue, Huflage et al. postulated that QIR level 2 is favorable for osseous tissue assessment in hip imaging^13^. All of the earlier studies on the subject have in common that the right use of QIR improved image quality due to higher CNR and overall lower noise^13,18,19^. Following that principle, it can be assumed that there is even more improvement potential in intrinsically noisier scans, for example, when using dedicated low-dose scan protocols^4^. In our study, it was possible to increase the CNR by 25% (comparing Q1 to Q4), which theoretically could be used for dose reduction by applying a higher QIR level to a lower dose scan. Besides applying more contrast agent or using virtual monoenergetic reconstructions, the implementation of QIR seems to be another option to reduce radiation dose without compromising image quality. Further studies are necessary to investigate this aspect.

To draw the right conclusions from whatever body region is examined, reproducible absolute CT attenuation numbers are imperative. Therefore, it is important to mention that the different QIR levels affected the absolute attenuation values of vessels and the surrounding soft tissue in our study. However, the actual effect was less than 1% when comparing Q1 and Q4, which should be negligible in the vast majority of clinical scenarios.

Besides QIR, the selection of the right convolution kernel contributes to better image quality. Studies could demonstrate that the use of a sharper kernel for image reconstruction leads to overall improved image quality^20^, to a better visualization of vascular stents^21^ as well as to improved diagnostic accuracy^22^. The fact that image quality improvement through QIR is most pronounced when using sharper kernels suggests that both can be beneficial in synergistic fashion. It needs to be mentioned that noise and artifacts are generally removed via iterative reconstruction at the cost of a loss of details and amplified image blurring. However, in the case of PCD-CTA reconstructed with QIR, this did not have a negative impact on image quality. In our analysis of the blur metric, QIR had different effects on soft and sharp kernels. Whereas images reconstructed with the soft Bv48 kernels did not become blurrier with higher QIR strength, sharp Bv76 reconstructions suffered from an increase in blurring. Interestingly, the “blurrier” images were still subjectively preferred, potentially due to their less noisy appearance.

Several limitations of this single-center study must be acknowledged. Due to the experimental nature of the study, only five body donors (ten lower extremities) were examined with a single PCD-CT scanner from one manufacturer. Since we limited ourselves to maximum dose scans in order to minimize dose-dependent differences in image quality, the absolute influence of QIR may be different at lower scan doses with more intrinsic image noise. A direct comparison of QIR with conventional iterative reconstruction or filtered back projection was not possible for technical reasons. A standard scan protocol with 120 kV, ultra-high resolution mode and a slice thickness/increment of 1.0 mm was used to optimize comparability to existing studies. In addition, no further post-processing algorithms such as virtual monoenergetic reconstructions were used.

## Conclusion

Quantum iterative reconstruction of photon-counting CT angiographies has a positive influence on their image quality in terms of image noise and CNR. In particular, reconstructions with sharp convolution kernels benefit quantitatively from higher QIR levels. While the subjectively preferred QIR level is dependent on kernel selection, on average QIR level 3 provided the best objective and subjective image quality results over all reconstruction settings.

## Electronic supplementary material

Below is the link to the electronic supplementary material.

Supplementary Material 1

## Data Availability

The datasets generated and/or analyzed during the current study are not publicly available but are available from the corresponding author on reasonable request.

## Abbreviations

CNR: Contrast-to-noise-ratio
CTA: Computed tomography angiography
HU: Hounsfield units
PCD-CT: Photon-counting detector CT
QIR: Quantum Iterative Reconstruction
SD: Standard deviation
