# Supplementary material for: Combined influence of quantum iterative reconstruction level and kernel sharpness on image quality in photon counting CT angiography of the upper leg
Source: Sci Rep. 2024 Nov 13;14:27774. doi: 10.1038/s41598-024-79188-3 (PMC11561153; doi:10.1038/s41598-024-79188-3)
Supplement: Supplementary file 1 — Supplementary Material 1 [file 41598_2024_79188_MOESM1_ESM.docx]

**
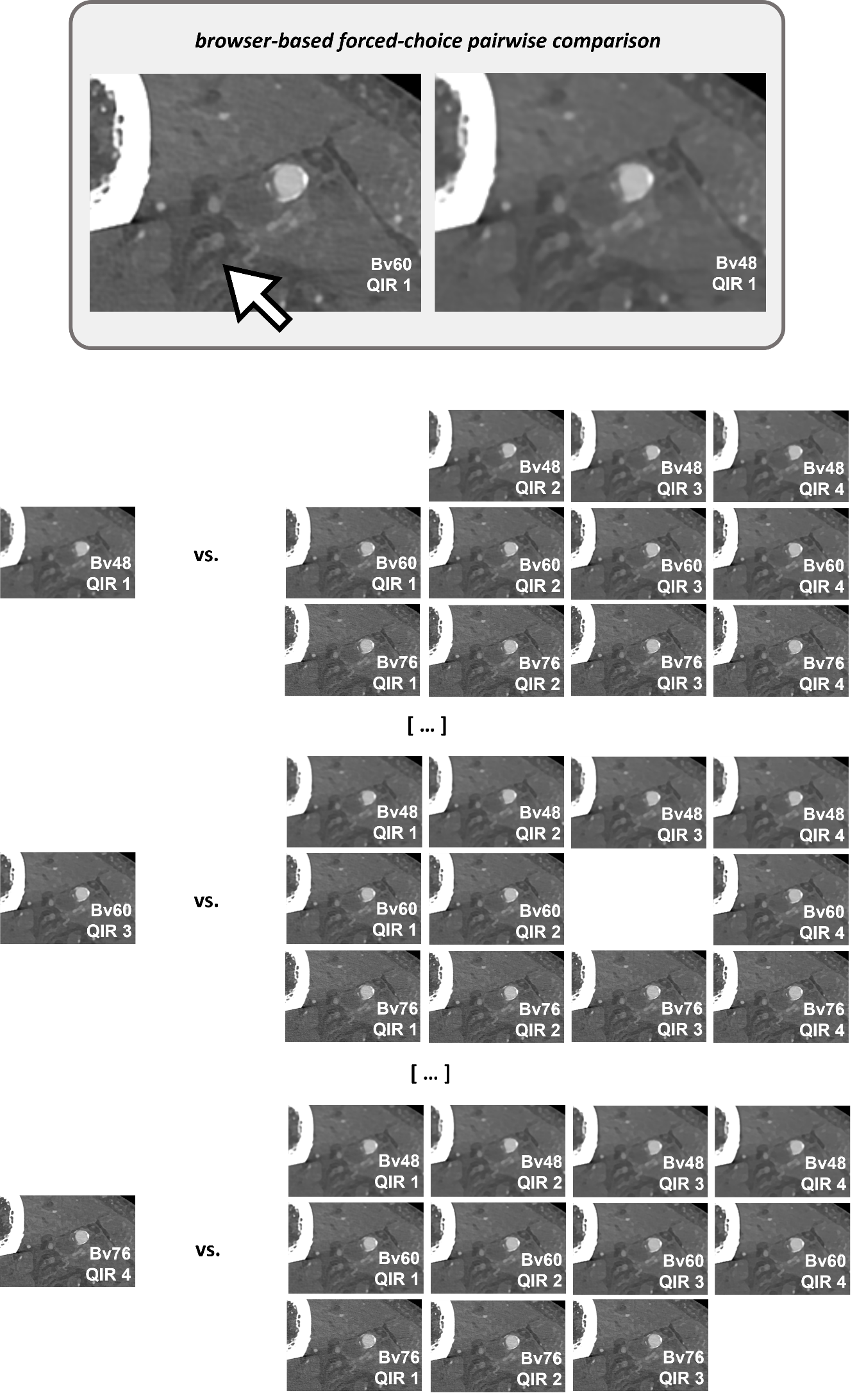
Supplementary Figure S1 – Schematically depiction of the decision-making process using the forced-choice pairwise comparison software.**

*The software randomly displays images of the same axial slice of the same extremity of the same body donor to the rater. Images of all possible combinations of convolution kernel and quantum iterative reconstruction level (12 individual images) are displayed as pairs resulting in 66 pairs (12 x (12-1)/2 = 66) per thigh. By clicking, the rater selects the subjectively better image. Based on this selection, the software generates an image quality ranking from 1 (best) to 12 (worst).*

*Bv = body vascular; QIR = quantum iterative reconstruction.*
